# Maintaining validity of neuropathic pain screening tools over time: challenges and opportunities

**DOI:** 10.1186/s41687-026-01086-1

**Published:** 2026-06-04

**Authors:** Giulia Mesaroli, Anthony V. Perruccio, Kristen M. Davidge, Jennifer Stinson

**Affiliations:** 1 Institute of Health Policy, Management and Evaluation, University of Toronto, 155 College Street, Toronto, Canada; 2 Child Health Evaluative Sciences, Research Institute, The Hospital for Sick Children, 686 Bay Street, Toronto, Canada; 3 Department of Rehabilitation Services, The Hospital for Sick Children, 555 University Ave Rm S229, Toronto, ON M5G 1X8 Canada; 4 Department of Physical Therapy, University of Toronto, 500 University Avenue, Toronto, Canada; 5 Dalla Lana School of Public Health, University of Toronto, 155 College Street, Toronto, Canada; 6 Schroeder Arthritis Institute, University Health Network, 522 University Ave Suite, Toronto, Canada; 7 Division of Plastic and Reconstructive Surgery, The Hospital for Sick Children, 555 University Ave, Toronto, Canada; 8 Department of Surgery, University of Toronto, 149 College Street, Toronto, Canada; 9 Lawrence S. Bloomberg Faculty of Nursing, University of Toronto, 155 College Street, Toronto, Canada

**Keywords:** Validity, Screening, Measurement properties, Psychometric properties

## Abstract

**Background:**

Significant efforts are made to validate screening tools at the time of development. This paper outlines the challenge in maintaining validity of neuropathic pain (NP) screening tools alongside advances in how NP condition is defined, diagnosed, and classified. To illustrate this, the validity of the Leeds Assessment of Neuropathic Symptoms and Signs (LANSS) was critiqued in relation to advances in knowledge of NP.

**Methods:**

This narrative review used a systematic literature search to identify content validity (including tool development) and criterion validity studies of the LANSS. Content validity (including face validity) was critiqued using a subset of COSMIN standards. Criterion validity was critiqued using a subset of QUADAS-2 standards.

**Results:**

Twelve papers were identified (nine pertain to criterion validity and three pertain to both content and criterion validity). Content validity studies with patients provided limited evidence of the tool’s relevance and comprehensibility. At face value, two items may no longer be relevant to NP. Six of 12 criterion validity studies may no longer be applicable (included patients who would no longer be classified with NP and/or the diagnostic assessment is outdated); six remaining studies reported variable results (sensitivity 22–86%, specificity 37–100%).

**Conclusion:**

Additional content validity testing of the LANSS is warranted to determine if the tool is relevant and comprehensible, and to determine whether revisions are needed to reflect the current state of knowledge of NP. Screening tool validity is not static over time; validation is a continuous process that may include tool revision.

**Supplementary Information:**

The online version contains supplementary material available at 10.1186/s41687-026-01086-1.

## Introduction

Screening tools can aid in identifying people who may have a particular condition and facilitate access to diagnostic assessment and treatment [1]. These tools are only useful if they are valid, an assessment typically made at the time of tool development [2]. Over time, changes in how a condition is defined, classified, or diagnosed can limit the validity of existing tools. This can be particularly challenging in fields where knowledge of a condition is in the early phases or evolving, or with conditions that are not observable on diagnostic tests (e.g., imaging, bloodwork) and rely on subjective report of symptoms (e.g., mental illness, pain) [2]. Continuously assessing screening tool validity can be resource intensive and may result in the need for tool revision. This is a significant methodological challenge that is not often addressed in the literature.

Since the inception of several screening tools for neuropathic pain (NP) in the early 2000s, there have been significant changes in how NP is defined, classified and diagnosed. The review aimed to critically discuss the challenges in maintaining the validity of NP screening tools alongside this evolving knowledge. To summarize literature on this broad topic and deepen our understanding, a narrative review is appropriate [3, 4]. To illustrate this challenge, the validity of the Leeds Assessment of Neuropathic Symptoms and Signs (LANSS), a self-report screening tool for NP, was critiqued [5]. The purpose of the LANSS is to discriminate between people with NP from people with other types of pain, for example, musculoskeletal or inflammatory pain. The LANSS was chosen as it was the first developed tool, recommended by the International Association for the Study of Pain (IASP) [6], and has been the most studied [7]. Both the original (LANSS) and self-report version (s-LANSS) were examined.

## Background

### Challenge in the field of measurement

The most important measurement properties of a screening tool are content and criterion validity (Table 1). Content validity, including face validity, refers to the relevance, comprehensiveness and comprehensibility of a tool [8]. This is established during tool development and through formal testing with patients and clinicians [8]. During development, the condition and population need to be clearly defined and reflect the current state of knowledge to ensure the tool’s relevance [2, 10]. If the definition of a condition changes, there may be a conceptual mismatch between an existing screening tool and the contemporary definition. This mismatch can limit the tool’s relevance (includes items that are no longer relevant) or comprehensiveness (important items are missing). These limitations may be readily apparent (face validity) or require formal content validity testing to determine whether tool revision is needed. Content validity is the most important property; if the tool is not relevant or comprehensive, it will compromise other properties including criterion validity [10].

**Table 1:** Taxonomy of relevant measurement terms

Measurement term	Definition
Content validity	The degree to which the items in a tool form the construct to be measured, typically pertaining to relevance (to the construct, population, and context of use), comprehensiveness and comprehensibility [8].
Face validity	The degree to which items in the tool look as though they form the construct to be measured. Considered an aspect of content validity [2, 8].
Criterion validity	The degree to which the scores of a tool adequately reflect a reference test [8].
Reference test	A test considered to be a sound indication of whether or not a condition is truly present. Also known as a gold standard or criterion standard test [9].
Sensitivity	Proportion of patients with the condition that have a positive test result [9].
Specificity	Proportion of patients without the condition that have a negative test result [9].
False positive	Proportion of patients without the condition with a positive test result [9].
False negative	Proportion of patients with the condition with a negative test result [9].

Criterion validity refers to the match between the results of a screening tool and a diagnostic assessment, typically expressed using sensitivity (SE) and specificity (SP) estimates [8]. When testing criterion validity, the classification and diagnostic assessment of patients with the condition should reflect the current state of knowledge [2, 9, 11, 12]. Over time, if a new classification system is adopted and/or a new diagnostic assessment is considered gold standard, results (SE, SP) from prior studies may no longer be applicable in the context of the contemporary understanding of the condition. In this case, criterion validity would need to be re-tested.

### Challenge in the field of neuropathic pain

The LANSS, developed in 2001, included five self-report and two clinician-report items [5]. A modified version (s-LANSS) was developed in 2005 (7 self-report items) [5]. Since their inception, the definition, classification and diagnostic approach to NP have changed, raising concern as to whether the tools are currently valid.

When the LANSS was developed, NP was defined as pain “caused by a lesion or dysfunction in the nervous system.” [13] Pain arising from nervous system trauma (e.g., surgery), disease (e.g., multiple sclerosis), and dysfunction (e.g., complex regional pain syndrome (CRPS)) were conceptualized as NP. Later in 2011, the IASP published a new definition of NP, “pain caused by a lesion or disease of the somatosensory nervous system,” removing the term ‘dysfunction’ [14]. In 2016, a new pain mechanism was introduced, nociplastic pain, to include pain arising from nervous system dysfunction [15]. As such, pain conditions like CRPS were no longer considered a type of NP, but rather nociplastic pain. From a diagnostic lens, the first diagnostic criteria were introduced in 2008 [16] and then updated in 2016 [17], highlighting the importance of a thorough subjective history (including identifying a relevant neurological disease/trauma), conducting a sensory examination (to confirm sensory changes correspond to a neuroanatomical distribution), and diagnostic tests confirming a neurological lesion/disease. Lastly, in 2019, the International Classification of Diseases (ICD-11), introduced the first classification system of NP, solidifying that conditions arising from nervous system dysfunction, like CRPS, are no longer classified as NP [18].

## Methods

This is a narrative literature review; this methodology was chosen as it is flexible and allows synthesis of literature on a broad, complex topic that requires a nuanced description [3, 4]. A systematic literature search was used to ensure all relevant studies of content or criterion validity of the LANSS were identified. The validity of the LANSS was critiqued in relation to the current state of knowledge of NP, specifically, the contemporary 2011 definition [14], 2016 diagnostic grading system [17], and the ICD-11 classification [18]. With content validity studies, reference is made to study publications years and the definition, diagnosis and classification of NP that was available at that time of publication.

### Literature search

Searches were conducted by a medical librarian in MEDLINE, Embase, and CINAHL from inception to March 10, 2025, limited to English-language peer-reviewed papers (Supplemental File 1). Search terms included terms of NP, screening, LANSS, and a measurement property filter [19].

Inclusion criteria were: (1) the study pertains to the English-language version of the LANSS and (2) study describes content (including tool development) and/or criterion validity. Two team members screened titles and abstracts and reviewed full-texts for eligibility using Covidence [20]. A third team member resolved discrepancies.

### Data extraction

A data extraction spreadsheet was developed to extract data pertaining to study characteristics, content, face and criterion validity (described below). This spreadsheet was piloted and refined. Two team members independently rated the validity of each study and tool (described below). Discrepancies in ratings were resolved through discussion with a third team member. Data were synthesized in narrative and tabular format.

### Content, face and criterion validity

Content validity studies were critiqued using the COnsensus-based Standards for the selection of health Measurement INstruments (COSMIN V2.0) manual [10]. Two team members independently evaluated six of 68 COSMIN study quality standards. Five standards applied to tool development studies and one to content validity studies (Table 2). The remaining 62 standards were not relevant to this review and pertained to risk of bias concerns (e.g., sample size, recording interviews). The six standards applied were narratively described, to allow for a nuanced description and critique, in line with the narrative review methodology. Further, categorical ratings of standards in the COSMIN manual were not appropriate for application in this review. For example, standard #1 (Table 2) regarding the definition of the condition, is categorically rated as clearly described/not described [21]. Although a study may have clearly described the condition, it may not align with the contemporary definition, and therefore the categorical ratings did not capture this methodological challenge.

**Table 2:** COSMIN standards applied to tool development and content validity studies

**Standards applied to tool development studies to ensure relevance of the tool**
1. Is a clear description provided of the construct to be measured?
2. Is the origin of the construct clear: was a theory, conceptual framework or disease model used or clear rationale provided to define the construct to be measured?
3. Is a clear description provided of the target population for which the tool was developed?
4. Is a clear description provided of the context of use?
5. Was the tool development study performed in a sample representing the target population for which the tool was developed?
**Standards applied to content validity studies to evaluate relevance, comprehensibility or comprehensiveness of the tool**
6. Was testing performed in a sample representing the target population?

Standards 1–5 were obtained from COSMIN [10] section 5.1 (#1–4) and 6.1 (#1) regarding tool development studies

Standard 6 was obtained from COSMIN [10] section 6.1 (#11) regarding a pilot study using a draft version of the tool to evaluate comprehensibility. This standard was deemed relevant and applied to all content validity studies (using a draft or final version of the tool) evaluating any aspect of content validity (relevance, comprehensiveness, or comprehensibility)

Face validity of the LANSS was evaluated independently by two pain physicians using eight of 10 COSMIN standards for good content validity (Supplemental File 2) [10]. Evaluating face validity is especially recommended if a tool was developed a long time ago, patients were not adequately involved in tool development and/or there are limited content validity studies [10]. Two of 10 standards were not rated as they were intended for patient evaluation via content validity testing. As per the manual, each of the eight standards were categorically rated (sufficient/insufficient/unclear) and pooled for an overall rating of relevance, comprehensiveness, and comprehensibility of the LANSS.

Criterion validity studies were evaluated using the Quality Assessment of Diagnostic Accuracy Studies (QUADAS-2) manual [12]. First, two team members independently rated three of seven QUADAS-2 standards that pertained to whether the patients, screening tool, and reference test are applicable to the contemporary understanding of NP (Table 3). Standards were categorically rated as low, high or unclear concern. The remaining four standards were not relevant to this review and pertained to risk of bias concerns (e.g., blinding).

**Table 3:** QUADAS-2 standards applied to criterion validity studies

Applicability standard	Rating options (level of concern) and description
**Patient selection:** Is there concern that the included patients do not match the review question?	**Low** = Description of included patients suggests they would currently be classified with NP (ICD-11 classification of NP) [18] **High** = Description of included patients indicates they would not currently be classified with NP (e.g., patients with CRPS) **Unclear** = Insufficient description of included patients
**Screening tool:** Is there concern that the screening tool [LANSS], its conduct, or interpretation differ from the review question?	**Low** = Physician’s judgement of the LANSS at face value indicates sufficient relevance, comprehensibility, and comprehensiveness. **High** = Physician’s judgement of the LANSS at face value indicates insufficient relevance or comprehensiveness or comprehensibility. **Unclear** = Physician’s judgement of the LANSS at face value indicates one or more aspects (relevance, comprehensibility and comprehensiveness) is of unclear sufficiency but all other aspects are sufficient.
**Reference test:** Is there concern that the target condition [NP] as defined by the reference test does not match the review question?	**Low** = An acceptable reference test was used, defined as a clinical diagnosis using the contemporary definition of NP [14] with/without the 2016 IASP Diagnostic grading system [17] **High** = An acceptable reference test was not used (e.g., clinical diagnosis using an outdated definition of NP, self-report diagnosis, screening tool) **Unclear** = Insufficient description of the reference test

Standards were obtained from QUADAS-2 Standards 1b, 2b, and 3b [12]

Description of each rating option was developed for this paper, as recommended in the QUADAS-2 manual

ICD-11 = International Classification of Diseases, 11^th^ edition; LANSS = Leeds Assessment of Neuropathic Symptoms and Signs; NP = neuropathic pain

Review question: To critically appraise the criterion validity of the LANSS, a screening tool for neuropathic pain

## Results

**Fig. 1:**
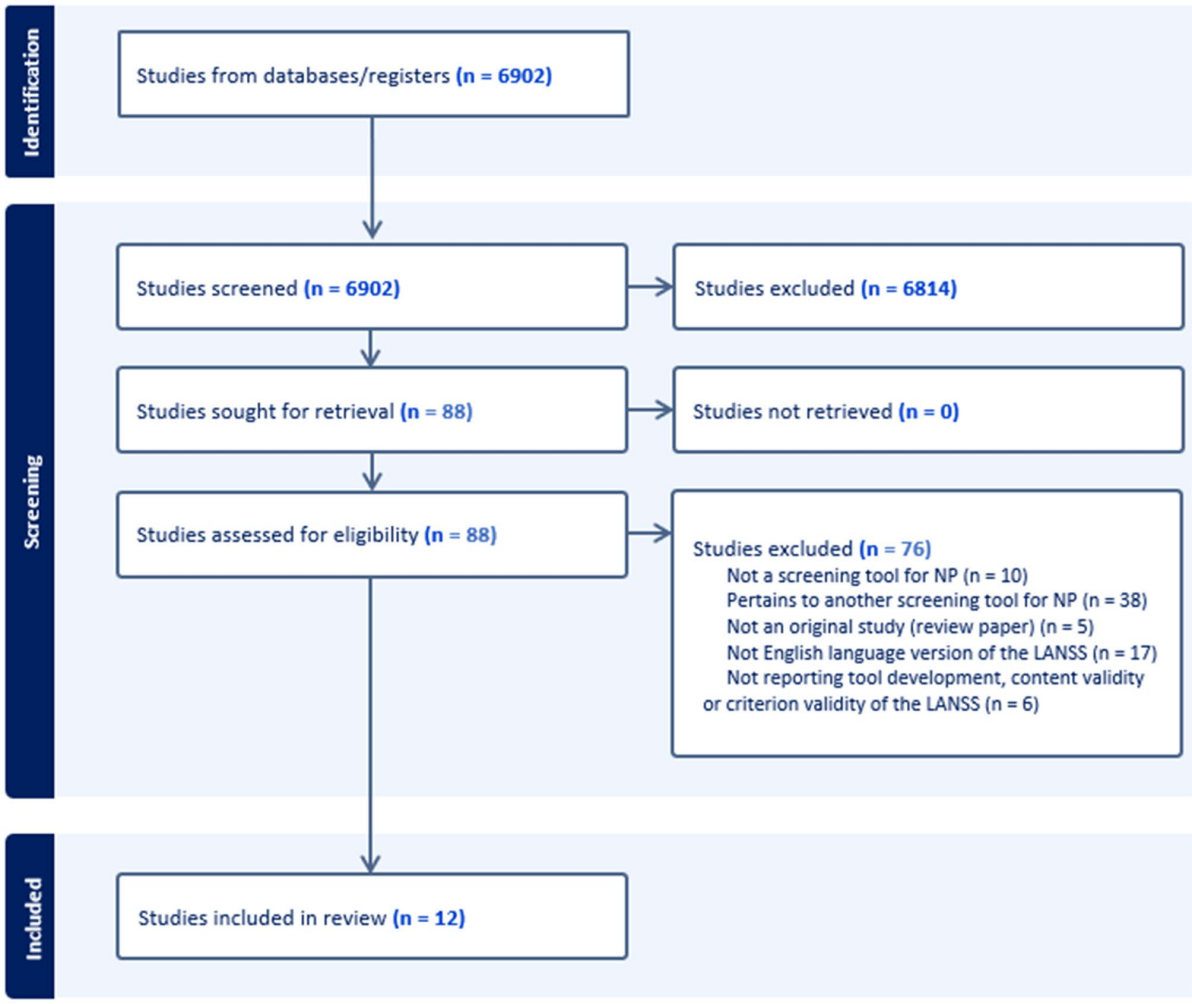
PRISMA flow diagram

Twelve papers were included (Fig. 1); nine papers tested criterion validity, and three papers pertain to content and criterion validity. Table 4 describes study characteristics.

**Table 4:** Characteristics of included studies (*N* = 12)

Characteristic	Number of studies, n (%)
**Study setting**^a^	
Cancer clinic	2 (17%)
Community-based	1 (8%)
Pain clinic	4 (33%)
Surgical clinic	5 (42%)
Orthopaedic clinic	1 (8%)
**Country**	
Australia	3 (25%)
Canada	1 (8%)
United Kingdom	6 (50%)
United States of America	2 (17%)
**Study design**^b^	
Content validity studies (*n* = 2)	
Qualitative interviews	2 (17%)
Criterion validity studies (*n* = 12)	
Cohort study	3 (25%)
Cross-sectional	8 (67%)
Interventional	1 (8%)

^a^1 study recruited patients from more than 1 setting, total > 100%

^b^Study design was not described for tool development studies

### Content validity

One paper described development of the LANSS and tested item relevance [5], one paper described development of the s-LANSS [22], and one paper tested the comprehensibility of the s-LANSS [23].

#### Tool development

The 2001 development paper of the LANSS clearly defined NP, citing the 1994 IASP definition (COSMIN#1–2; Table 2) [5]. Although this was applicable at the time, this differs from the 2011 definition. The target population (COSMIN#3) was described as people with NP, no sub-type of NP (e.g., cancer-related) was specified. This paper also described content validity testing in patients with diverse types of NP and patients with CRPS, implying the tool was intended for broad use and for patients with CRPS. The target population differs from the current understanding of NP, as CRPS is no longer classified as NP. The purpose of the tool (COSMIN#4) was clearly described, to distinguish NP from nociceptive pain. Although this was applicable at the time, the current classification includes a third mechanism, nociplastic pain [15]. Items were identified (COSMIN#5) by experts and patients; patients were not described and therefore it is unclear if they were representative of patients meeting the contemporary definition of NP. The outdated definition of NP, target population, and the tool’s purpose, limit the relevance of the tool to the contemporary understanding of NP.

The 2005 development paper of the s-LANSS described that two clinician-reported items were reworded for self-report [22]. No details were provided on how revisions were determined including whether or not patients were consulted regarding comprehensibility (COSMIN#5).

#### Content validity testing

The development study of the LANSS asked patients in interviews to rate the relevance of items (yes/no) in a draft version of the tool (*N* = 60) [5]. With respect to the representativeness of patients (COSMIN#6), three patients had CRPS and would not currently be classified with NP [23]. This is a small proportion (5%), and may have minimally impacted selection of relevant items. This paper provided limited details on results; the number of items eliminated and descriptive statistics of item relevance (%yes) was not reported.

A study of the LANSS interviewed patients with cancer (*N* = 125) to test item comprehensibility [23]. All patients appeared to be representative of NP (COSMIN#6) as they all had cancer-related NP, currently classified as NP. Results indicated 4/7 items were not comprehended by a small proportion of patients (*n* = 6, <5%).

#### Face validity

At face value, both versions of the LANSS were equally rated using COSMIN standards for good content validity (Supplemental File 2); differences between versions did not result in differences in ratings.

The overall relevance of the LANSS was rated as insufficient because two items were not entirely relevant to NP. One item (color changes) was considered a symptom of CRPS and only one sub-type of NP (erythromelalgia), not relevant to the spectrum of NP. One item pertained to both skin temperature changes and the pain quality burning; although burning was considered relevant, skin temperature changes were considered relevant to CRPS and not NP. These two items represent a large portion of the tool (18% of items and 25% of the total score), hence rating the tool’s relevance as insufficient.

The comprehensiveness was rated as sufficient as no important symptoms of NP were deemed to be missing. The comprehensibility was rated as insufficient because two of seven items were confusing and the response options for all seven items did not exactly match the question; these items and responses represented a significant portion of the tool.

#### Criterion validity

Twelve criterion validity studies were included (Table 5); seven tested the LANSS, four tested the s-LANSS, and one tested both versions. The screening tool was rated as highly concerning in all studies, as the relevance and comprehensibility of the LANSS were rated insufficient at face value with respect to the contemporary definition of NP.

**Table 5:** QUADAS-2 applicability concerns of criterion validity studies (*N* = 12 studies)

Study author and publication year	Definition of NP	Reference Standard	Applicability Concerns			SE %	SP %
			Patient Selection	Screening Tool	Reference Test		
**LANSS (original version)**							
Bennett 2001 [5]	1994	Physician diagnosis	✓	✗	✗	85	80
Potter et al. 2003 [23]	1994	Physician diagnosis	✓	✗	✗	79	100
Tampin et al. 2013 [24]	2011	IASP Grading System	✓	✗	✓	22	88
Sadler et al. 2013 [25]	Unclear	Physician diagnosis	?	✗	?	76	94
Hardy et al. 2013 [26]	Other	Clinician diagnosis	✓	✗	✓	86	100
Markman et al. 2015 [27]	None	QTFCS	✓	✗	✓	38	75
Shamji et al. 2016 [28]	Other	Surgeon diagnosis	✓	✗	✓	71	96
Walker et al. 2024 [29]	2011	Physician diagnosis	✓	✗	✓	86	38
**s-LANSS (self-report version)**							
Bennett et al. 2005 [22]	1994	Physician diagnosis	✗	✗	✗	74	76
Weingarten et al. 2007 [30]^a^	None	Clinician diagnosis	?	✗	?	52–57	69–78
Moreton 2015 [31]^b^	None	painDETECT	✓	✗	✗	73	71
Tampin et al. 2019 [32]	2011	IASP Grading System	✓	✗	✓	24	97
Walker et al. 2024 [29]^c^	2011	Physician diagnosis	✓	✗	✓	75–81	37–48

Applicability concerns: ✓ = Low concern; ? = Unclear concern; ✗ = High concern

Definition: 2011 = 2011 IASP definition [14] and 1994 = 1994 IASP definition [33]

SE = Sensitivity; SP = Specificity; QTFCS = Quebec Task Force Classification System; IASP = International Association for the Study of Pain; LANSS = Leeds Assessment of Neuropathic Symptoms and Signs

^a^Two administration modes tested (mail and telephone); results differ by administration mode

^b^ Used an alternate cut-off score (>9) rather than the original cut-off score (≥12)

^c^ Two administration modes tested (clinician interview and independent self-report), results differ by administration mode

Four studies were highly concerning regarding the applicability of patients and/or reference test to the current understanding of NP [5, 22, 23, 31]. Criterion validity of the LANSS (SE 85%, SP 80%) and s-LANSS (SE 74%, SP 76%) were first tested in 2001 and 2005, respectively [5, 22]. In both studies, the reference test was a clinical diagnosis related to the 1994 definition of NP (high concern) and a significant portion (20%) of patients had CRPS and would not currently be classified with NP (high concern) [5, 22]. Two studies (one of the LANSS and one of the s-LANSS) were rated as low concern regarding patients, but high concern regarding the reference test [23, 31]. Two studies (one of the LANSS and one of the s-LANSS) did not sufficiently describe patients or the reference test; both were rated as unclear concern [25, 30].

Six studies (four of the LANSS, one of the s-LANSS, and one of both versions) were rated as low concern regarding patients and the reference test as the studies provided sufficient description to determine alignment with the contemporary classification and diagnosis of NP [24, 26–29, 32]. Results reported in these studies (SE 22–86%, SP 37–100%) were highly variable [24, 26–29, 32].

## Discussion

The challenge of maintaining NP screening tool validity over time has been exemplified with a narrative review of the LANSS in the face of changes in the classification and diagnosis of NP. This review identified 12 studies of the LANSS, all studies examined criterion validity, and three also examined content validity. Content validity studies with patients provided limited evidence of the tool’s relevance and comprehensibility [5, 22, 23]. At face value, two items may no longer be relevant to the contemporary understanding of NP. Six of 12 criterion validity studies may no longer be applicable to the contemporary understanding of NP as they included patients who would no longer be classified with NP and/or the diagnostic assessment is outdated [5, 22, 23, 25, 30, 31]. Six remaining criterion validity studies reported variable results (sensitivity 22–86%, specificity 37–100%) [24, 26–29, 32]. This highlighted opportunities to address this methodological challenge for the LANSS and more broadly in the field of measurement.

### Challenge with NP screening tools

With NP, the definition, classification and diagnosis became more restrictive over time [14, 17, 18]. This type of change has specific implications for the validity of NP screening tools. Tools may include items that are no longer relevant to NP, limiting the tool’s relevance. With the LANSS, two items appeared irrelevant at face value and are particularly problematic as they contribute significantly to the score. This has negative implications for criterion validity, as patients without NP may score positively on a tool by endorsing irrelevant items, lowering the tool’s SP. This was evident in one study of the LANSS that linked poor SP (37%) to a high proportion of patients with CRPS with a false positive score [29]. Clinically, with a SP of 37%, the false positive rate is unreasonably high (63%), meaning 63% of patients who do not have NP will screen positively and potentially undergo unnecessary treatment for NP or unnecessarily be referred to pain specialists. This poses a burden both to the individual and to the health care system. From a research lens, NP screening tools are often used to estimate prevalence rates of NP [34, 35], if the false positive rate is high, this will overestimate the prevalence rate of NP.

Re-evaluating the content validity of the LANSS is warranted, and potentially other NP screening tools as well. With the LANSS, qualitative interviews with a diverse group of patients and clinicians are needed to explore the relevance of items in the tool to the contemporary understanding of NP, and to determine how to address potentially irrelevant items (e.g., deletion, revision) [21]. If items are deleted, item scores and the cut-off score would need to be re-established. This re-evaluation is ideally sequential, where the content validity of the current tool should be evaluated, followed by revisions if necessary, and lastly, re-evaluation of the criterion validity using the contemporary IASP diagnostic criteria as a gold-standard test [17].

### Challenge in the broader field of measurement

In contrast to the example with NP, if the definition, classification, and diagnosis of a condition broadens over time, existing screening tools may be missing relevant items, limiting comprehensiveness. Patients with the condition may score negatively (false negative) if their symptoms are not included in the tool, with the effect of lowering SE estimates. This scenario is likely more problematic than when a definition becomes more restrictive, as screening tools ideally are highly SE (as opposed to diagnostic tools that have a balanced profile of SE and SP) [9]. This occurred when three symptoms were added to the diagnostic criteria of post-traumatic stress disorder (PTSD), for example [36]. In response, three items were added to the PTSD Checklist [37].

There may be scenarios where changes in knowledge of a condition may impact on a tool’s required recall period. For example, symptom duration is a diagnostic criteria for depression which informs the 2-week recall period of the Patient Health Questionnaire-9 depression screener [38]. There may be scenarios where cultural or linguistic changes impact screening tool validity. For example, the McGill Pain Questionnaire [39], developed in 1975, includes the pain descriptor ‘lancinating’, which is no longer commonly used. Another example is with the increasing popularity of electronic administration, the Colored Pain Drawing, initially developed for paper-administration, is difficult to reproduce electronically [40].

### Opportunities

A number of steps can be taken to maintain validity of existing screening tools when there are significant advances in knowledge of a condition. First, the relevance and comprehensiveness of the tool should be re-examined at face value. In a straightforward example, as with PTSD where three explicit diagnostic criteria were added [36], it may be sensical to immediately revise the tool. In a more nuanced case, as with NP, a literature review of the tool’s validity is warranted to evaluate whether existing evidence is applicable in the context of the contemporary understanding of the condition. This can aid in identifying gaps in validity and help determine whether further content validity testing is needed to inform tool revision or criterion validity testing is needed using a new diagnostic assessment (after content validity is established).

Developing a new tool is extremely resource intensive (costly and time-intensive) and should be reserved only in cases where existing tools require major revision. Developing a new tool involves input from diverse stakeholders and typically includes multiple study phases to determine the scope of the tool, generate items, reduce items, determine a scoring mechanism and pilot testing to refine the language [2, 21]. When possible, efforts should be made to revise existing tools (e.g., by adding or deleting items, revising the recall period or scoring mechanisms), followed by re-evaluating criterion validity of the revised version against the contemporary diagnostic test.

The COSMIN [10] and QUADAS-2 [12] manuals can aid in evaluating existing validation studies. However, these manuals in their current format do not fully acknowledge this methodological challenge and systematic reviews that use these manuals may not readily identify these limitations in published studies. There are opportunities for these manuals to better address this challenge in future versions. The QUADAS-2 [12] manual could explicitly prompt users to consider whether the diagnosis or patient classification used in the study is contemporary or outdated. For outdated tools, the COSMIN [10] manual suggests prioritizing the evaluation of face validity and ignoring results from outdated content validity studies. Instead of ignoring outdated studies, the manual could prompt users to consider the extent to which study results are outdated; for example, by considering the proportion of participants that would no longer be classified as having the condition. Second, categorical ratings for some study quality standards need to be modified to make them applicable to studies that used an outdated conceptualization of the condition. Third, standards regarding patient representativeness could be applied to all content validity studies, not only development and pilot studies. Last, with item relevance, the manual could prompt users to consider not only the proportion of irrelevant items, but also the proportion of the overall score attributable to the irrelevant items.

There is also opportunity for scientific communities to acknowledge this challenge such as when publishing new guidelines on screening or diagnosis. This can prompt scientists to conduct further validity testing and prompt clinicians to consider whether legacy tools used in practice are still appropriate. With NP, the Neuropathic Pain Special Interest Group of the IASP, which was involved in the updated definition, classification and diagnosis of NP, has the opportunity to commission a systematic review of existing NP screening tools to determine if existing tools are still valid or if a new tool is warranted.

### Limitations

This was not a systematic review to evaluate the validity of the LANSS, therefore study quality was not comprehensively evaluated. The LANSS may require additional testing because of risk of bias concerns in addition to applicability concerns. This review was restricted to the English-language version of the LANSS and thus results are not generalizable to other versions.

## Conclusion

The validity of neuropathic pain screening tools is not static, but subject to change over time with changes in the definition, classification or diagnostic approach to neuropathic pain. Continuous evaluation and modification of screening tools can be a methodological challenge. This is a present challenge for the LANSS, where additional testing is needed to evaluate the tool’s relevance to the contemporary understanding of NP and determine if revisions are needed.

## Electronic supplementary material

Below is the link to the electronic supplementary material.

Supplementary Material 1

Supplementary Material 2

## Data Availability

Not applicable
