# Supplementary material for: Maintaining validity of neuropathic pain screening tools over time: challenges and opportunities
Source: J Patient Rep Outcomes. 2026 Jun 4;10:139. doi: 10.1186/s41687-026-01086-1 (PMC13457814; doi:10.1186/s41687-026-01086-1)
Supplement: Supplementary file 1 [file 41687_2026_1086_MOESM1_ESM.docx]

**Supplemental File 1. MEDLINE Literature Search Strategy**

Ovid MEDLINE(R) ALL <1946 to March 10, 2025>

| **#** | **Searches** |
| --- | --- |
| 1 | Neuralgia/ or (neuralg* or "nerve pain?" or neuropath* or "postherpetic neuralg*" or "trigeminal neuralg*" or erythermalgia* or erythromelalgia* or sciatica or neurodynia*).tw,kf. |
| 2 | *mass screening/ or *"Pain Measurement"/mt or ((Screening or assessment or measurement or classification* or "sensory descri*" or "neuropathic pain") adj3 (tool or instrument? or checklist? or survey? or questionnaire? or measur*)).tw,kf. |
| 3 | 1 and 2 |
| 4 | ("ID Pain" or DN4 or "Douleur Neuropathique 4" or painDETECT or "promis neuropathic pain quality " or lanss or "leeds assessment of neuropathic symptoms and signs" or "neuropathic pain questionnaire").tw,kf. |
| 5 | 3 or 4 |
| 6 | (instrumentation or methods).sh. or ("Validation Studies" or "Comparative Study").pt. or exp "psychometrics"/ or (psychometr* or clinimetr* or clinometr* or "outcome measure*").tw,kw. or "outcome assessment (health care)"/ or "outcome assessment"/ or "observer variation"/ or ("observer variation" or reproducib* or reliab* or unreliab* or valid* or "coefficient of variation" or coefficient or homogeneity or homogeneous or "internal consistency").tw,kw. or "Health Status Indicators"/ or "reproducibility of results"/ or "discriminant analysis"/ or (cronbach* and (alpha or alphas)).ti,ab. or (item and (correlation* or selection* or reduction*)).ti,ab. or (agreement or precision or imprecision or "precise values" or test-retest).tw,kw. or (test and retest).ti,ab. or (reliab* and (test or retest)).ti,ab. or (stability or interrater or inter-rater or intrarater or intra-rater or intertester or inter-tester or intratester or intra-tester or interobserver or inter-observer or intraobserver or intra-observer or intertechnician or inter-technician or intratechnician or intra-technician or interexaminer or inter-examiner or intraexaminer or intra-examiner or interassay or inter-assay or intraassay or intra-assay or interindividual or inter-individual or intraindividual or intra-individual or interparticipant or inter-participant or intraparticipant or intra-participant or kappa or kappa's or kappas or repeatab*).tw,kw. or ((replicab* or repeated) and (measure or measures or findings or result or results or test or tests)).tw,kw. or (generaliza* or generalisa* or concordance).tw,kw. or (intraclass and correlation*).ti,ab. or (discriminative or "known group" or "factor analysis" or "factor analyses" or "factor structure" or "factor structures" or dimension* or subscale*).ti,ab. or ((multitrait and scaling and (analysis or analyses)) or ("item discriminant" or "interscale correlation*" or error or errors or "individual variability" or "interval variability" or "rate variability")).ti,ab. or (variability and (analysis or values)).ti,ab. or ((uncertainty and (measurement or measuring)) or ("standard error of measurement" or sensitiv* or responsive*)).ti,ab. or (limit and detection).ti,ab. or ("minimal detectable concentration" or interpretab*).ti,ab. or ((minimal or minimally or clinical or clinically) and (important or significant or detectable) and (change or difference)).ti,ab. or (small* and (real or detectable) and (change or difference)).ti,ab. or ("meaningful change" or "ceiling effect" or "floor effect" or "Item response model" or IRT or Rasch or "Differential item functioning" or DIF or "computer adaptive testing" or "item bank" or "cross-cultural equivalence" or development* or develop*).ti,ab. |
| 7 | 5 and 6 |
| 8 | limit 7 to english language |
| 9 | remove duplicates from 8 |

Note: The MEDLINE search strategy was translated for Embase and CINAHL by a medical research librarian
