# Supplementary material for: Maintaining validity of neuropathic pain screening tools over time: challenges and opportunities
Source: J Patient Rep Outcomes. 2026 Jun 4;10:139. doi: 10.1186/s41687-026-01086-1 (PMC13457814; doi:10.1186/s41687-026-01086-1)
Supplement: Supplementary file 2 [file 41687_2026_1086_MOESM2_ESM.docx]

**Supplemental File 2. COSMIN standards for good content validity applied to the LANSS**

| **STANDARD** | **CATEGORICAL RATING OPTIONS**  (+) sufficient (-) insufficient (?) unclear | **PHYSICIANS RATING** |
| --- | --- | --- |
| **RELEVANCE** | | |
| 1. Are the items relevant for the construct (*neuropathic pain, NP*)? | (+) ≥85% of items relevant to the construct  (-) <85% of items relevant to the construct  (?) unclear | - |
| 2. Are the items relevant for the target population (*people with NP*)? | (+) ≥85% of items relevant to the target population  (-) <85% of items relevant to target population  (?) unclear | - |
| 3. Are the items relevant for the context of use (*screening*)? | (+) ≥85% of the items relevant to the context of use  (-) <85% of items relevant to the context of use  (?) unclear | ? |
| 4. Are the response options appropriate? | (+) ≥85% of the response options appropriate  (-) <85% of response options are appropriate  (?) unclear | - |
| 5. Is the recall period appropriate? | (+) recall period is appropriate  (-) recall period is inappropriate  (?) unclear | + |
| ***Overall relevance rating*** | (+) Standard 1 and 2 are (+) and 2 other criteria are (+)  (-) Standard 1 or 2 are (-)  (?) At least one of Standard 1 or 2 are (?)  (+/- indeterminate) All other situations | **Insufficient** |
| **COMPREHENSIVENESS** | | |
| 6. Are all key concepts included? | (+) no key concepts are missing  (-) key concepts are missing  (?) unclear | + |
| ***Overall comprehensiveness rating*** | Same rating is applied from Standard 6 | **Sufficient** |
| **COMPREHENSIBILITY** | | |
| 7. Are the items appropriately worded? | (+) ≥85% of the items and response options appropriately worded  (-) >15% of items or response options are not appropriately worded  (?) unclear | - |
| 8. Do the response options match the question? | (+) ≥85% of the response options match the questions  (-) >15% of response options do not match the question  (?) unclear | - |
| ***Overall comprehensibility rating*** | (+) Standards 7 and 8 are (+)  (-) Standard 7 and/or 8 are (-)  (?) Standard 7 and/or 8 are (?)  (+/- indeterminate) All other situations | **Insufficient** |

Standards are COSMIN[20] Criteria for Good Content Validity #1-6, 9-10. Rating options (+/ - / ?) and descriptions were obtained from the COSMIN manual. In this paper, physicians used these as a guide, in addition to their clinical judgements. LANSS = Leeds Assessment of Neuropathic Symptoms and Signs, original and self-report versions
